# Supplementary material for: Ocular conjunctival inoculation of SARS-CoV-2 can cause mild COVID-19 in rhesus macaques
Source: Nat Commun. 2020 Sep 2;11:4400. doi: 10.1038/s41467-020-18149-6 (PMC7467924; doi:10.1038/s41467-020-18149-6)
Supplement: Supplementary file 2 — Reporting Summary [file 41467_2020_18149_MOESM2_ESM.pdf]

nature research

Corresponding author(s): Chuan Qin

Last updated by author(s): Jul 18, 2020

Reporting Summary

Nature Research wishes to improve the reproducibility of the work that we publish. This form provides structure for consistency and transparency in reporting. For further information on Nature Research policies, see Authors & Referees and the Editorial Policy Checklist .

Statistics

For all statistical analyses, confirm that the following items are present in the figure legend, table legend, main text, or Methods section.

n/a	Confirmed	
☐	☒	The exact sample size (*n*) for each experimental group/condition, given as a discrete number and unit of measurement
☐	☒	A statement on whether measurements were taken from distinct samples or whether the same sample was measured repeatedly
☒	☐	The statistical test(s) used AND whether they are one- or two-sided<br>*Only common tests should be described solely by name; describe more complex techniques in the Methods section.*
☒	☐	A description of all covariates tested
☒	☐	A description of any assumptions or corrections, such as tests of normality and adjustment for multiple comparisons
☒	☐	A full description of the statistical parameters including central tendency (e.g. means) or other basic estimates (e.g. regression coefficient) AND variation (e.g. standard deviation) or associated estimates of uncertainty (e.g. confidence intervals)
☒	☐	For null hypothesis testing, the test statistic (e.g. *F*, *t*, *r*) with confidence intervals, effect sizes, degrees of freedom and *P* value noted<br>*Give P values as exact values whenever suitable.*
☒	☐	For Bayesian analysis, information on the choice of priors and Markov chain Monte Carlo settings
☒	☐	For hierarchical and complex designs, identification of the appropriate level for tests and full reporting of outcomes
☒	☐	Estimates of effect sizes (e.g. Cohen's *d*, Pearson's *r*), indicating how they were calculated

Our web collection on statistics for biologists contains articles on many of the points above.

Software and code

Policy information about availability of computer code

Data collection	All data were collected with GraphPad Prism 8.0 software (GraphPad Software Inc., San Diego, CA)
Data analysis	All data analysis were performed with GraphPad Prism 8.0 software (GraphPad Software Inc., San Diego, CA)

For manuscripts utilizing custom algorithms or software that are central to the research but not yet described in published literature, software must be made available to editors/reviewers. We strongly encourage code deposition in a community repository (e.g. GitHub). See the Nature Research guidelines for submitting code & software for further information.

Data

Policy information about availability of data

All manuscripts must include a data availability statement. This statement should provide the following information, where applicable:

- Accession codes, unique identifiers, or web links for publicly available datasets
- A list of figures that have associated raw data
- A description of any restrictions on data availability

The complete genome for this SARS-CoV-2 was submitted to NCBI (SARS-CoV-2/WH-09/human/2020/CHN, accession No. MT093631.2). All raw data are available from the corresponding author on reasonable request.

Field-specific reporting

Please select the one below that is the best fit for your research. If you are not sure, read the appropriate sections before making your selection.

☒ Life sciences ☐ Behavioural & social sciences ☐ Ecological, evolutionary & environmental sciences

Life sciences study design

All studies must disclose on these points even when the disclosure is negative.

Sample size	No statistical methods were used to predetermine sample size. For ethical considerations, the monkey size of animal experiments is small. Five male rhesus macaques (Macaca Mulatta) between the ages of 3 and 5 years were used in this study. The results of this animal experiment are qualitative, therefore, no sample size calculation was performed.
Data exclusions	No data were excluded from the analyses.
Replication	Five rhesus macaques were included in this study. For viral loads, viral titers, HE and IHC stain experiments, three independent experiments were repeated. All attempts at replication were successful.
Randomization	For the animal study, five animals were randomly selected to undergo the subsequent exposure. The tests were also randomly selected from all samples. The pictures were representatively shown.
Blinding	During the study, all monkeys and all samples were coded. The technicians did not know the information of the animals or samples.

Reporting for specific materials, systems and methods

We require information from authors about some types of materials, experimental systems and methods used in many studies. Here, indicate whether each material, system or method listed is relevant to your study. If you are not sure if a list item applies to your research, read the appropriate section before selecting a response.

Materials & experimental systems

n/a	Involved in the study	
☐	☒	Antibodies
☐	☒	Eukaryotic cell lines
☒	☐	Palaeontology
☐	☒	Animals and other organisms
☒	☐	Human research participants
☒	☐	Clinical data

Methods

n/a	Involved in the study	
☒	☐	ChIP-seq
☒	☐	Flow cytometry
☒	☐	MRI-based neuroimaging

Antibodies

Antibodies used

7D2 antibody for anti-SARS-CoV-2 S protein (mouse antibody, laboratory preparation, 1:200) for IHC, recombinant anti-Mouse IgG antibody (ab190475, Abcam, 1:1000) for IHC, goat-anti mouse IgG conjugated HRP (ZDR-5307, Beijing ZSGB Biotechnology, 1:200) for IHC, Spike protein of SARS-CoV-2 (Sino Biological, 40591-V08H) and goat-anti monkey IgG conjugated HRP (ab112767, Abcam, 1:10000) for ELISA.

Validation

Laboratory preparation of the antibody of SARS-CoV-2 Spike-1 (S1) protein
Mice were immunized with purified SARS-CoV-2 S1 protein (Sino biological) and splenocytes of hyper immunized mice were fused with myeloma cells. Positive clones were selected by ELISA using SARS-CoV-2 S1 protein. The cell supernatant of 7D2 clone, binding to SARS-CoV-2 S1 protein, was collected for immunofluorescence analysis.
7D2 antibody for anti-SARS-CoV-2 S protein (mouse antibody, laboratory preparation, 1:200)

A recombinant anti-Mouse IgG antibody [RM104] (ab190475, Abcam, 1:1000)
https://www.abcam.cn/mouse-igg-antibody-rm104-ab190475.html

Goat anti-mouse IgG secondary antibody conjugated HRP (ZDR-5307, Beijing ZSGB Biotechnology, 1:200)
http://www.zsbio.com/product/ZDR-5307

Spike protein of SARS-CoV-2 (Sino Biological, 40591-V08H)
https://cn.sinobiological.com/recombinant-proteins/2019-ncov-cov-spike-40591-v08h

Goat anti-monkey IgG secondary antibody conjugated HRP (ab112767, Abcam, 1:10000)
https://www.abcam.cn/goat-monkey-igg-hl-hrp-ab112767.html

Eukaryotic cell lines

Policy information about cell lines

Cell line source(s)	VERO C1008 [Vero 76, clone E6, Vero E6] (ATCC® CRL-1586™) were obtained from ATCC (CRL-1586).
Authentication	To follow the protocol provided by ATCC. None of the cell lines have been authenticated.
Mycoplasma contamination	The cell line tested negative for mycoplasma contamination.
Commonly misidentified lines (See ICLAC register)	There is no commonly misidentified cell lines used in this study.

Animals and other organisms

Policy information about studies involving animals; ARRIVE guidelines recommended for reporting animal research

Laboratory animals	Rhesus macaques (Macaca Mulatta), male, 3-5-year-old.
Wild animals	This study did not involve wild animals.
Field-collected samples	The study did not involve samples collected from the field.
Ethics oversight	All procedures in this study involving animals were reviewed and approved by the Institutional Animal Care and Use Committee of the Institute of Laboratory Animal Science, Peking Union Medical College (BLL20009).

Note that full information on the approval of the study protocol must also be provided in the manuscript.
