# Supplementary material for: Ocular conjunctival inoculation of SARS-CoV-2 can cause mild COVID-19 in rhesus macaques
Source: Nat Commun. 2020 Sep 2;11:4400. doi: 10.1038/s41467-020-18149-6 (PMC7467924; doi:10.1038/s41467-020-18149-6)
Supplement: Supplementary file 3 — Source Data [file 41467_2020_18149_MOESM3_ESM.pdf]

Supplementary information

Ocular conjunctival inoculation of SARS-CoV-2 can cause mild COVID-19 in rhesus macaques

Wei Deng[†,1], Linlin Bao[†,1], Hong Gao[†,1], Zhiguang Xiang[†,1], Yajin Qu[†,1], Zhiqi Song[†,1], Shunran Gong[1], Jiayi Liu[2], Jiangning Liu[1], Pin Yu[1], Feifei Qi[1], Yanfeng Xu[1], Fengli Li[1], Chong Xiao[1], Qi Lv[1], Jing Xue[1], Qiang Wei[1], Mingya Liu[1], Guanpeng Wang[1], Shunyi Wang[1], Haisheng Yu[1], Ting Chen[1], Xing Liu[1], Wenjie Zhao[1], Yunlin Han[1], Chuan Qin[*,1]

[1] Key Laboratory of Human Disease Comparative Medicine, Chinese Ministry of Health, Beijing Key Laboratory for Animal Models of Emerging and Remerging Infectious Diseases, Institute of Laboratory Animal Science, Chinese Academy of Medical Sciences and Comparative Medicine Center, Peking Union Medical College, Beijing, China.

[2] Department of Radiology, Bejing Anzhen Hospital, Capital Medical University, Beijing, China.

†These authors contributed equally to this work.

*Correspondence should be addressed to Chuan Qin, Email: qinchuan@pumc.edu.cn.

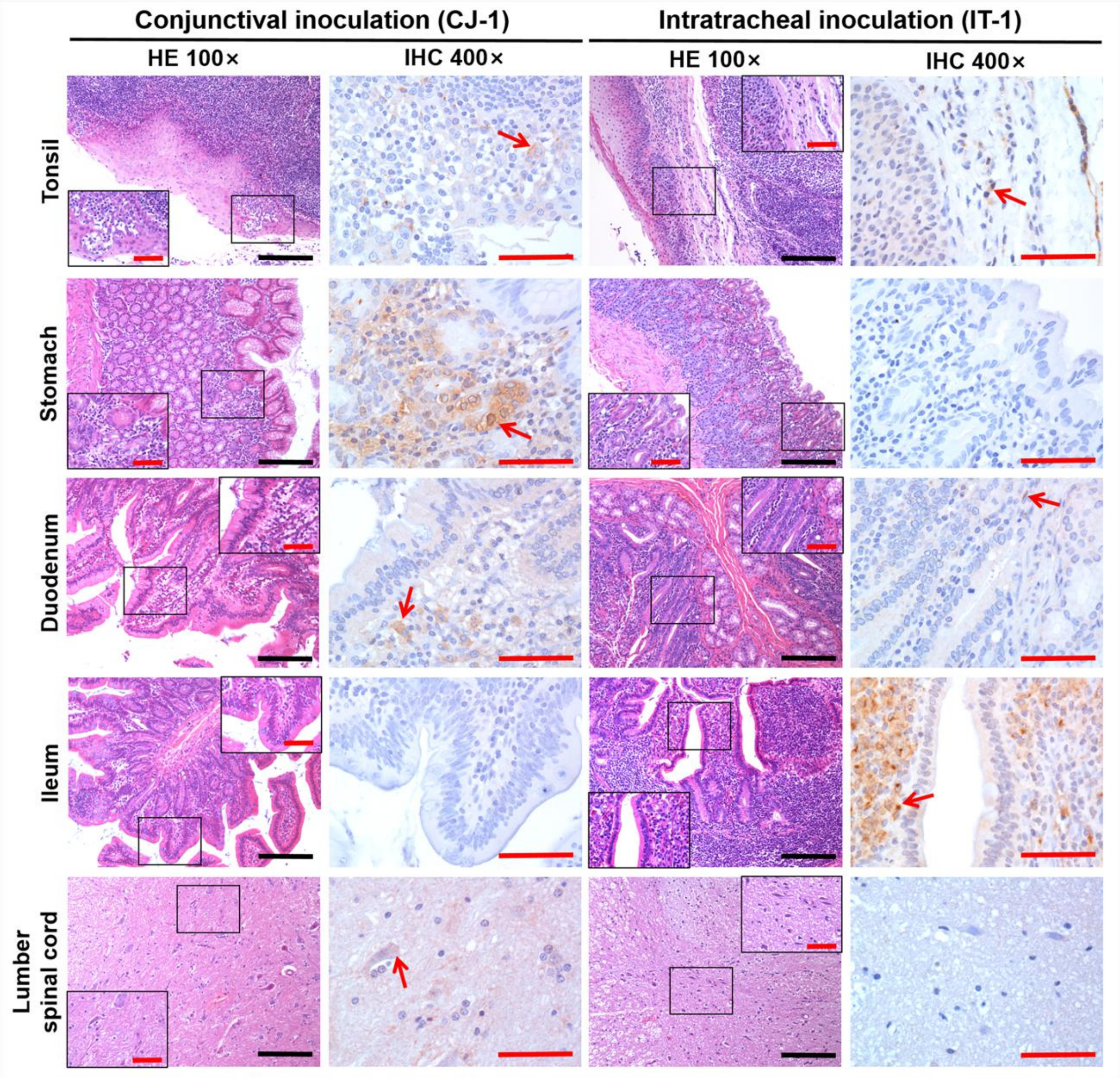

Supplementary Figure 1 Comparison of the viral distributions in the tonsil; alimentary tract, including the stomach, duodenum, and ileum; and lumber spinal cord in CJ-1 and IT-1.

Sequential sections were stained by H&E and subjected to IHC. The fields in the H&E images outlined in black are magnified. The IHC images show the same field outlined in black at 400× magnification. Black scale bar = 100 μm, red scale bar = 50 μm. Data are representative of three independent experiments.

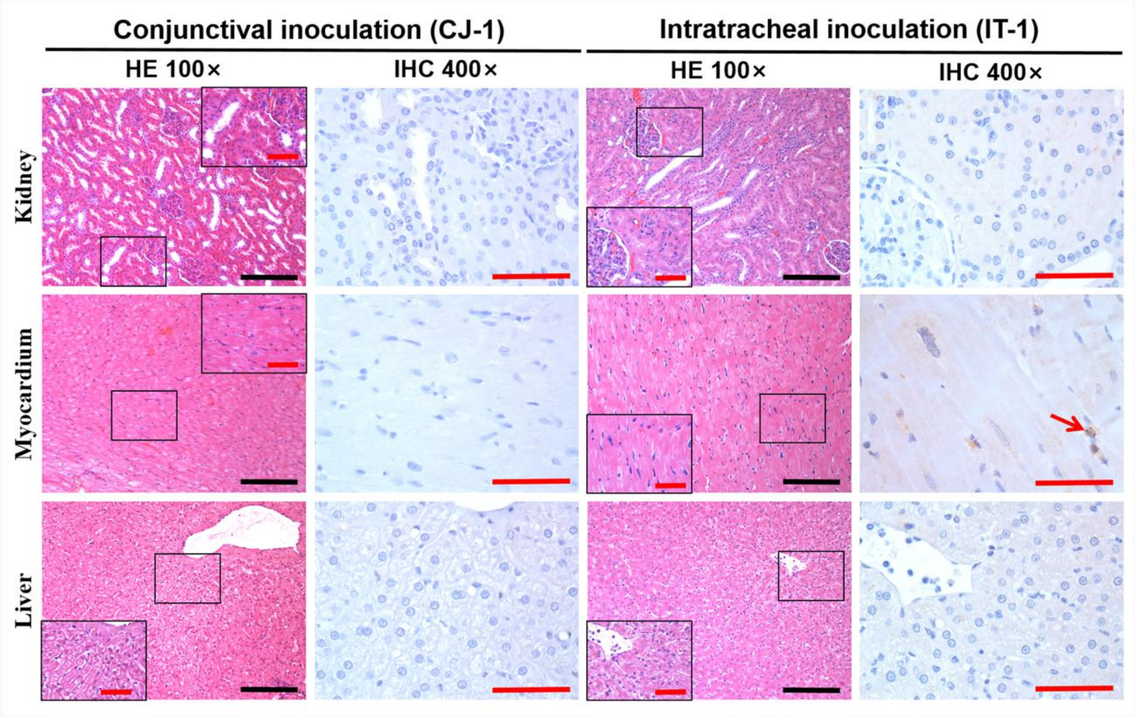

Supplementary Figure 2 Slight viral antigen staining was detected in the kidney, heart, and liver of IT-1, but the corresponding organs from CJ-1 were negative.

Sequential sections were stained by H&E and subjected to IHC. The fields in the H&E images outlined in black are magnified. The IHC images showed the same field outlined in black at 400× magnification. Black scale bar = 100 µm, red scale bar = 50 µm. Data are representative of three independent experiments.

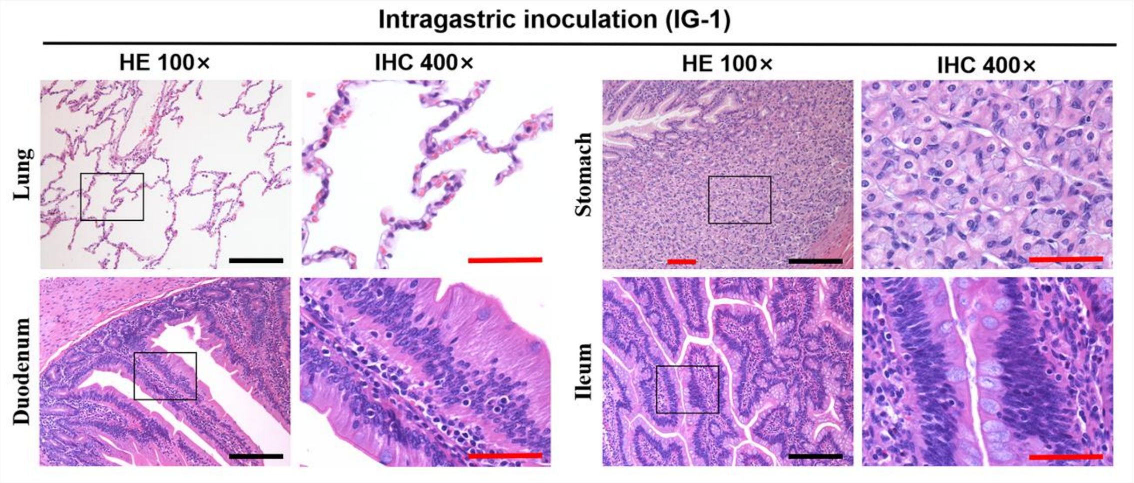

Supplementary Figure 3 No substantial histopathological changes were observed in tissues collected from IG-1.

The lung, stomach, duodenum, and ileum were stained with H&E. H&E-stained sections are shown at 100× magnification and magnified at 400× magnification (black frame). Black scale bar = 100 μm, red scale bar = 50 μm. Data are representative of three independent experiments.

Supplementary Table 1 Viral distributions in IG-1

Tissue	IG-1 (Log_{10} RNA copies/mL)
Cerebellum	0
Cerebrum	0
Lacrimal gland	0
Optic nerve	0
Conjunctiva	0
Nasal mucosa	0
Nasal turbinate	0
Nostril	0
Nasal septum	0
Cheek pouch	0
Parotid gland	0
Cervical cord	0
Thoracic spinal cord	0
Lumber spinal cord	0
Epiglottis	0
Soft palate	0
Trachea	0
Pulmonary lymph node	0
Hilar lymph nodes	0
Lung-lower left	0
Lung-lower right	0
Lung-middle left	0
Lung-middle right	0
Lung-right accessory	0
Lung-upper left	0
Lung-upper right	0

Stomach	0
Pancreas	0
Duodenum	0
Jejunum	0
Ileum	0
Colon	0
Rectum	0
Caecum	0
Intestinal lymph nodes	0
Pararectal lymph nodes	0
Kidney	0
Epididymis	0
Testis	0
Bladder	0
Mandibular lymph node	0
Thymus	0
Lingual tonsil	0
Pharyngeal tonsil	0
Cervical lymph nodes	0
Axillary lymph nodes	0
Inguinal lymph nodes	0
Spleen	0
Heart	0
Liver	0
Brown adipose tissue	0
